# Supplementary material for: Maternal Linoleic Acid Overconsumption Alters Offspring Gut and Adipose Tissue Homeostasis in Young but Not Older Adult Rats
Source: Nutrients. 2020 Nov 11;12(11):3451. doi: 10.3390/nu12113451 (PMC7697261; doi:10.3390/nu12113451)
Supplement: Supplementary file 1 [file nutrients-12-03451-s001.zip › Suppl Figures_Nutrients_Marchix et al.pdf]

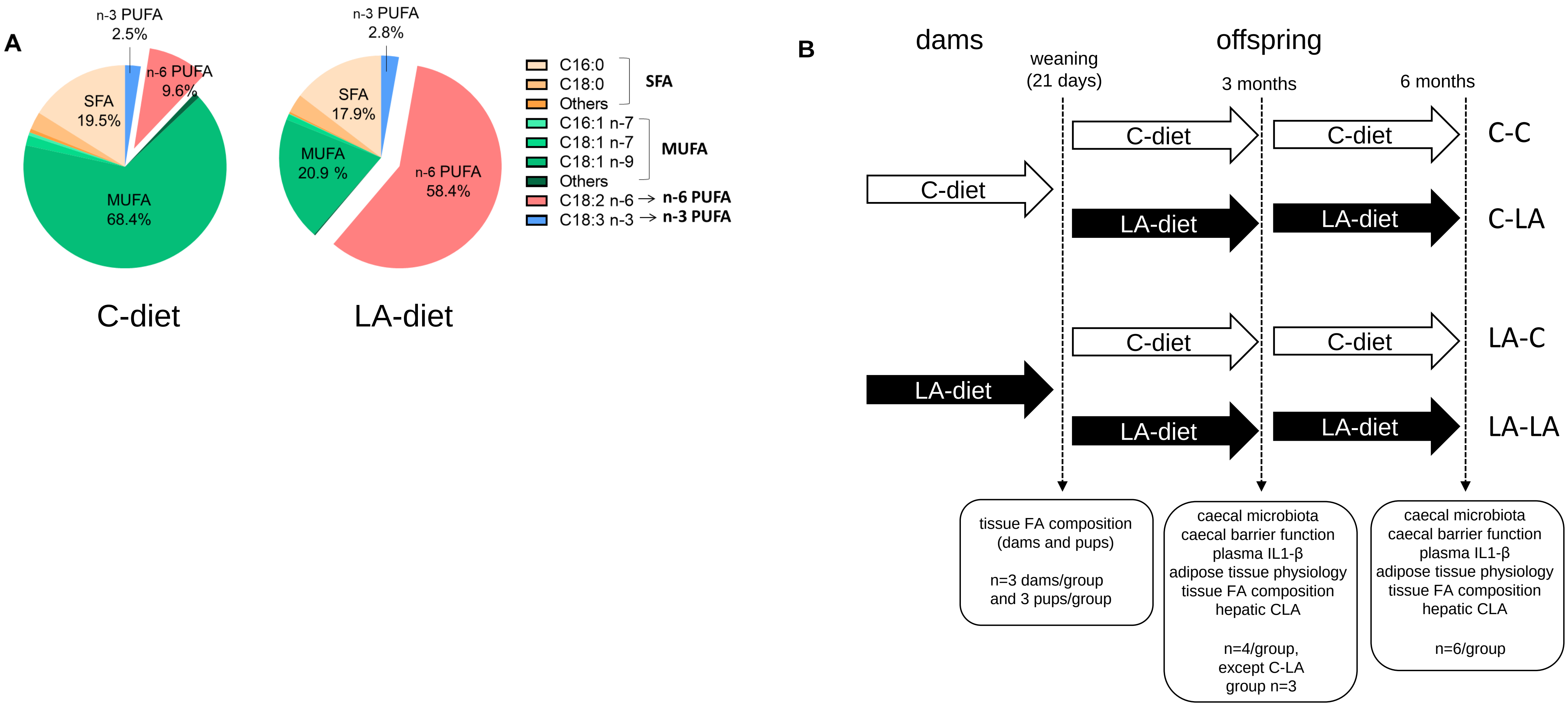

Supplemental Figure 1: A. Fatty acid composition of the experimental diets. B. Animal study design
CLA: conjugated linoleic acid, FA: fatty acid, MUFA: mono-unsaturated fatty acids, PUFA: poly-unsaturated fatty acids, SFA: saturated fatty acids

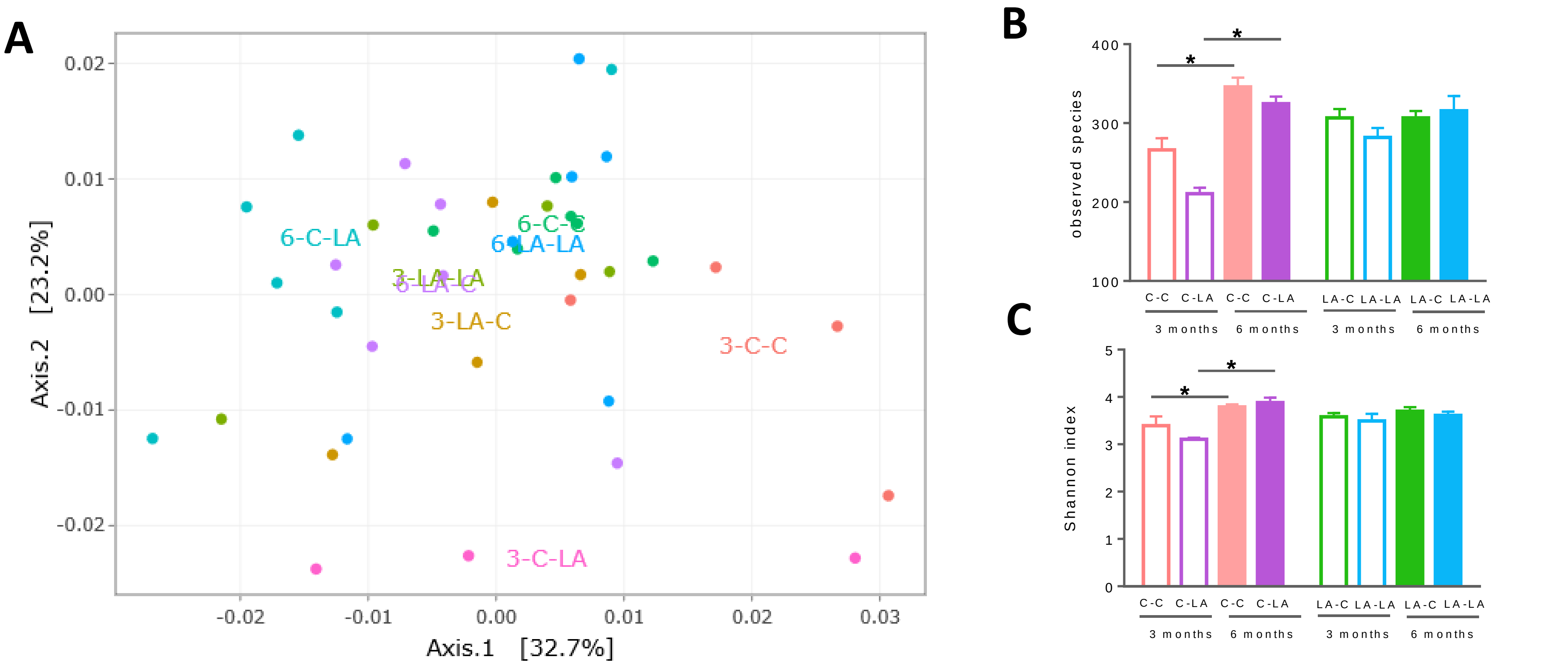

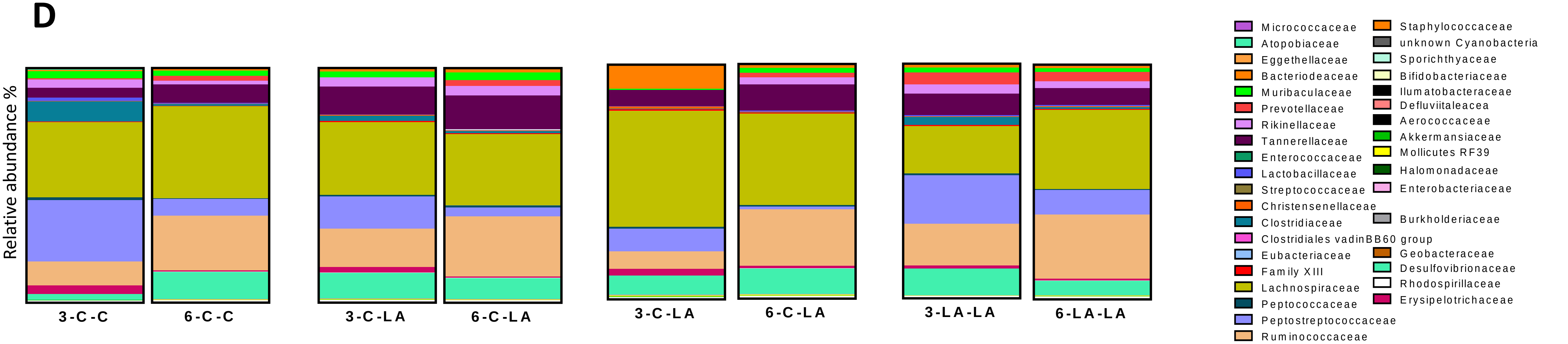

Supplemental Figure 2: A. PCoA based on Weight Unifrac distance of 3- and 6-month old rat microbiota. B. Microbiota richness of 3- and 6-month old rats. C. Microbiota diversity of 3- and 6-month old rats. D. Family relative abundance of 3- and 6-month old rat microbiota
