# Supplementary material for: Maternal Linoleic Acid Overconsumption Alters Offspring Gut and Adipose Tissue Homeostasis in Young but Not Older Adult Rats
Source: Nutrients. 2020 Nov 11;12(11):3451. doi: 10.3390/nu12113451 (PMC7697261; doi:10.3390/nu12113451)
Supplement: Supplementary file 1 [file nutrients-12-03451-s001.zip › Suppl Tables Marchix et al final.pdf]

Table S1: Primers used for PCR

Gene	FORWARD PRIMER	REVERSE PRIMER	ACCESSION NUMBER
ACTIN	CCCTAAGGCCAACCGTGAAA	CATACAGGGACAACACAGCCT	NM_031144.3
HPRT1	TAGGTCCATTCCTATGACTGTAGA	TGGCCTGTATCCAACACTTC	XM_008773659.2
RPS18	TCCACAGGAGGCCTACAC	CTCTTGGTGAGGTCAATGTCTG	XM_002727833.5
Fasn	TAAGCGGTCTGGAAAGCTGA	CACCAGTGTTTGTTCCTCGG	NM_017332.1
Lipe (HLS)	GTGTGTGAGCGCCTATTCAG	GCAGGCAATGGGGTCTTATG	NM_012859.1
LPL	ACTCAAAGTTAGGCCCAGCT	GGCCCAGCAACATTATCCAG	NM_012598.2
PPARg	TCAGCTCTTGTGAACGGGAT	GCACTGCCTATGAGCACTTC	NM_013124.3

Table S2: Fatty acid composition (% total fatty acids) of dam liver, mesenteric adipose tissue and mammary gland at weaning.

	Liver		Mesenteric fat		Mammary gland	
Maternal diet	C	LA	C	LA	C	LA
SFA	37.6 (1.9)	39.8 (0.5)	25.3 (0.3)	26.9 (0.2)*	32.0 (2.3)	37.1 (1.6)
MUFA	47.8 (3.4)	29.5 (4.5)*	59.1 (0.8)	33.0 (0.7)***	54.5 (1.4)	25.9 (1.5)***
n-6 PUFA	11.4 (4.2)	28.0 (3.7)**	14.4 (1.0)	38.8 (0.9)***	12.0 (1.0)	35.6 (0.2)***
18:2 n-6	*4.8 (1.6)*	*16.2 (1.3)**	*13.8 (1.0)*	*36.3 (1.0)****	*9.6 (1.4)*	*31.5 (0.8)****
20:4 n-6	*5.8 (2.4)*	*9.2 (3.6)*	*0.3 (0.0)*	*1.0 (0.1)****	*1.5 (0.4)*	*2.3 (0.4)*
n-3 PUFA	3.2 (1.2)	2.8 (0.9)	1.2 (0.1)	1.2 (0.0)	1.5 (0.1)	1.3 (0.0)*
n-6/n-3	3.6	11.71*	12.5	31.4***	8.1	28.3***

* P<0.05, ** P<0.01, *** P<0.001

Table S3: Fatty acid composition (% total fatty acids) of offspring liver and mesenteric adipose tissue at weaning.

	Liver		Mesenteric fat	
Maternal diet	C	LA	C	LA
SFA	40.7 (2.0)	35.3 (0.4)	37.6 (0.5)	35.6 (0.9)
MUFA	39.5 (0.8)	20.8 (2.1)**	54.5 (0.7)	28.9 (0.7)***
n-6 PUFA	13.7 (1.2)	38.8 (1.5)***	6.6 (0.2)	34.0 (1.5)***
18:2 n-6	*5.0 (0.3)*	*20.8 (0.4)****	*5.7 (0.1)*	*31.5 (1.5)****
20:4 n-6	*7.4 (0.6)*	*13.4 (1.0)***	*0.6 (0.1)*	*1.3 (0.1)***
n-3 PUFA	5.8 (0.5)	5.0 (0.4)	1.2 (0.1)	1.4 (0.1)
n-6/n-3	2.4	7.8***	5.7	24.8***

* P<0.05, ** P<0.01, *** P<0.001

Table S4: Microbiota family relative abundances in the rat caecum at 3 months of age

	C-C		C-LA		LA-C		LA-LA		Maternal diet	Weaning diet	Maternal x weaning diet
Actinobacteria, Bifidobacteriaceae	0.11	(0.05)	0.00	(0.00)	0.00	(0.00)	0.00	(0.00)	ns	ns	ns
Actinobacteria, Sporichthyaceae	0.01	(0.00)	0.01	(0.00)	0.00	(0.00)	0.00	(0.00)	0.01	ns	ns
Actinobacteria, Micrococcaceae	0.18	(0.04)	0.21	(0.05)	0.21	(0.08)	0.51	(0.27)	ns	ns	ns
Actinobacteria, Atopobiaceae	0.55	(0.32)	0.02	(0.01)	0.03	(0.01)	0.02	(0.01)	ns	ns	ns
Actinobacteria, Eggethellaceae	0.09	(0.02)	0.07	(0.01)	0.07	(0.02)	0.06	(0.02)	ns	ns	ns
Bacteroidetes, Bacteriodeaceae	0.65	(0.27) a	10.15	(3.72) b	1.43	(0.57) a	1.22	(0.18) a	0.02	0.009	0.007
Bacteroidetes,Muribaculaceae	2.93	(1.02)	0.66	(0.20)	2.47	(0.33)	2.15	(0.10)	ns	0.05	ns
Bacteroidetes,Prevotellaceae	0.54	(0.37)	0.00	(0.00)	0.03	(0.02)	5.21	(2.42)	ns	ns	ns
Bacteroidetes,Rikinellaceae	3.59	(1.76)	0.01	(0.01)	3.99	(2.40)	3.84	(0.54)	ns	ns	ns
Bacteroidetes,Tannerellaceae	4.22	(1.67)	6.84	(2.02)	11.99	(2.23)	9.22	(2.07)	0.03	ns	ns
Firmicutes, Enterococcaceae	0.02	(0.01)	0.00	(0.00)	0.01	(0.01)	0.01	(0.01)	ns	ns	ns
Firmicutes Lactobacillaceae	1.46	(0.25) a	0.21	(0.04) b	0.22	(0.07) b	0.53	(0.23) b	0.04	0.03	0.002
Firmicutes Streptococcaceae	0.03	(0.02)	0.03	(0.00)	0.02	(0.01)	0.08	(0.04)	ns	ns	ns
Firmicutes Christensenellaceae	0.22	(0.05) a	0.96	(0.09) b	0.39	(0.12) a	0.25	(0.09) a	0.01	0.008	0.007
Firmicutes Clostridiaceae	8.64	(1.61) a	0.39	(0.15) b	2.29	(0.75) b	3.53	(1.49) b	ns	0.02	0.003
Firmicutes Clostridiales vadinBB60 group	0.01	(0.01)	0.00	(0.00)	0.01	(0.01)	0.00	(0.00)	ns	ns	ns
Firmicutes Defluviitaleacea	0.02	(0.02)	0.00	(0.00)	0.00	(0.00)	0.02	(0.01)	ns	ns	ns
Firmicutes Eubacteriaceae	0.01	(0.01)	0.03	(0.01)	0.01	(0.01)	0.00	(0.00)	ns	ns	0.03
Firmicutes Family XIII	0.41	(0.11)	0.61	(0.04)	0.57	(0.21)	0.43	(0.14)	ns	ns	ns
Firmicutes Lachnospiraceae	32.00	(4.80) a	49.46	(3.28) b	31.07	(1.69) a	20.17	(2.77) a	0.001	ns	0.002
Firmicutes Peptococcaceae	1.26	(0.47)	0.83	(0.25)	0.73	(0.11)	0.86	(0.17)	ns	ns	ns
Firmicutes Peptostreptococcaceae	26.23	(4.66)	9.82	(7.99)	13.67	(2.80)	20.68	(3.91)	ns	ns	0.03
Firmicutes Ruminococcaceae	10.13	(3.01)	7.53	(0.94)	16.48	(2.81)	17.78	(3.20)	0.01	ns	ns
Firmicutes Erysipelotrichaceae	3.78	(1.61)	2.80	(0.15)	2.35	(0.29)	1.49	(0.18)	ns	ns	ns
Proteobacteria, Rhodospirillaceae	0.04	(0.01)	0.01	(0.01)	0.11	(0.05)	0.08	(0.02)	0.05	ns	ns
Proteobacteria, Desulfovibrionaceae	2.43	(1.67)	8.10	(2.84)	10.99	(3.35)	11.30	(1.04)	0.03	ns	ns
Proteobacteria, Desulfuromonadaceae	0.01	(0.01)	0.00	(0.00)	0.01	(0.01)	0.01	(0.01)	ns	ns	ns
Proteobacteria, Geobacteraceae	0.02	(0.01)	0.01	(0.01)	0.03	(0.02)	0.01	(0.00)	ns	ns	ns

Proteobacteria, Burkholderiaceae	0.15	(0.04)	0.50	(0.13)	0.32	(0.08)	0.22	(0.08)	ns	ns	0.02
Proteobacteria, Enterobacteriaceae	0.01	(0.01)	0.02	(0.01)	0.01	(0.01)	0.01	(0.00)	ns	ns	ns
Proteobacteria, Halomonadaceae	0.01	(0.01)	0.01	(0.00)	0.01	(0.01)	0.00	(0.00)	ns	ns	ns
Tenericutes, Mollicutes RF39	0.02	(0.01) a	0.37	(0.04) b	0.27	(0.13) ab	0.12	(0.02) ab	ns	ns	0.007
Verrucomicrobia, Akkermansiaceae	0.19	(0.09)	0.33	(0.06)	0.16	(0.08)	0.06	(0.01)	0.05	ns	ns

a,b,c: P<0.05

Table S5: Caecal microbiota family relative abundances at 6 months of age

	C-C		C-LA		LA-C		LA-LA		Maternal diet	Weaning diet	Maternal x weaning diet
Actinobacteria. Bifidobacteriaceae	0.01	(0.00)	0.00	(0.00)	0.00	(0.00)	0.03	(0.02)	ns	ns	ns
Actinobacteria. Sporichthyaceae	0.00	(0.00)	0.00	(0.00)	0.00	(0.00)	0.00	(0.00)	ns	ns	ns
Actinobacteria. Micrococcaceae	0.24	(0.06)	0.35	(0.10)	0.55	(0.10)	0.53	(0.18)	0.05	ns	ns
Actinobacteria. Atopobiaceae	0.00	(0.00)	0.02	(0.00)	0.03	(0.01)	0.02	(0.00)	ns	ns	ns
Actinobacteria. Eggethellaceae	0.03	(0.01)	0.07	(0.03)	0.05	(0.02)	0.06	(0.01)	ns	ns	ns
Bacteroidetes. Bacteriodeaceae	0.95	(0.08)	1.53	(0.22)	1.20	(0.17)	1.05	(0.19)	ns	ns	0.05
Bacteroidetes.Muribaculaceae	2.38	(0.30)	3.39	(0.45)	2.01	(0.41)	1.64	(0.21)	0.007	ns	ns
Bacteroidetes.Prevotellaceae	2.07	(0.29)	2.56	(0.45)	2.15	(0.36)	4.04	(1.16)	ns	ns	ns
Bacteroidetes.Rikinellaceae	1.71	(0.35)	4.01	(1.50)	3.08	(1.00)	3.02	(0.66)	ns	ns	ns
Bacteroidetes.Tannerellaceae	7.82	(0.84) ab	14.49	(3.15) a	11.13	(1.36) ab	7.14	(1.26) b	ns	ns	0.01
Firmicutes. Enterococcaceae	0.01	(0.01)	0.01	(0.01)	0.02	(0.01)	0.01	(0.00)	0.01	ns	ns
Firmicutes Lactobacillaceae	0.39	(0.08)	0.22	(0.05)	0.58	(0.11)	0.65	(0.11)	0.002	ns	ns
Firmicutes Streptococcaceae	0.02	(0.00)	0.04	(0.01)	0.05	(0.01)	0.05	(0.02)	ns	ns	ns
Firmicutes Christensenellaceae	0.22	(0.03)	0.36	(0.09)	0.38	(0.08)	0.34	(0.09)	ns	ns	ns
Firmicutes Clostridiaceae	0.73	(0.28)	0.88	(0.58)	0.11	(0.09)	0.80	(0.71)	ns	ns	ns
Firmicutes Clostridiales vadinBB60 group	0.07	(0.03)	0.03	(0.01)	0.00	(0.00)	0.05	(0.02)	ns	ns	0.03
Firmicutes Defluviitaleacea	0.03	(0.01)	0.02	(0.02)	0.01	(0.01)	0.02	(0.01)	ns	ns	ns
Firmicutes Eubacteriaceae	0.01	(0.00)	0.01	(0.01)	0.02	(0.01)	0.01	(0.00)	ns	ns	ns
Firmicutes Family XIII	0.31	(0.06)	0.46	(0.10)	0.43	(0.12)	0.56	(0.26)	ns	ns	ns
Firmicutes Lachnospiraceae	39.20	(0.59)	30.36	(1.60)	39.00	(4.25)	33.66	(2.88)	ns	0.02	ns
Firmicutes Peptococcaceae	0.26	(0.05)	1.02	(0.43)	0.62	(0.09)	0.47	(0.08)	ns	ns	ns
Firmicutes Peptostreptococcaceae	7.21	(2.30)	3.74	(1.99)	1.33	(0.68)	10.46	(3.61)	ns	ns	0.02
Firmicutes Ruminococcaceae	23.46	(1.22)	25.70	(4.41)	24.04	(2.23)	27.38	(5.00)	ns	ns	ns
Firmicutes Erysipelotrichaceae	0.45	(0.09)	0.55	(0.19)	1.11	(0.21)	0.89	(0.27)	0.02	ns	ns
Proteobacteria. Rhodospirillaceae	0.19	(0.06)	0.18	(0.03)	0.16	(0.05)	0.12	(0.04)	ns	ns	ns
Proteobacteria. Desulfovibrionaceae	11.60	(2.88)	8.96	(2.64)	10.88	(2.17)	6.21	(1.23)	ns	ns	ns
Proteobacteria. Desulfuromonadaceae	0.00	(0.00)	0.00	(0.00)	0.00	(0.00)	0.00	(0.00)	ns	ns	ns

Proteobacteria. Geobacteraceae	0.00	(0.00)	0.01	(0.01)	0.00	(0.00)	0.00	(0.00)	ns	ns	ns
Proteobacteria. Burkholderiaceae	0.21	(0.05)	0.29	(0.06)	0.28	(0.04)	0.12	(0.02)	ns	ns	0.02
Proteobacteria. Enterobacteriaceae	0.01	(0.01)	0.03	(0.01)	0.02	(0.01)	0.05	(0.03)	ns	ns	ns
Proteobacteria. Halomonadaceae	0.00	(0.00)	0.00	(0.00)	0.00	(0.00)	0.00	(0.00)	ns	ns	ns
Tenericutes. Mollicutes RF39	0.19	(0.05)	0.22	(0.14)	0.57	(0.20)	0.40	(0.21)	ns	ns	ns
Verrucomicrobia. Akkermansiaceae	0.02	(0.01)	0.06	(0.02)	0.12	(0.06)	0.13	(0.08)	ns	ns	ns

a,b,c P<0.05
